# Diabetes Mellitus and Prevalence of Other Comorbid Conditions: A Systematic Review

**DOI:** 10.7759/cureus.49374

**Published:** 2023-11-24

**Authors:** Harsh Bodke, Vasant Wagh, Gauri Kakar

**Affiliations:** 1 Community Medicine, Jawaharlal Nehru Medical College, Datta Meghe Institute of Higher Education and Research, Wardha, IND; 2 Medicine, Jawaharlal Nehru Medical College, Datta Meghe Institute of Higher Education and Research, Wardha, IND

**Keywords:** human immunodeficiency virus, urinary tract infections, tuberculosis, chd, dkd, sepsis, diabetes mellitus

## Abstract

Diabetes mellitus (DM) is a chronic metabolic disorder characterized by elevated blood glucose levels, severity continues to rise worldwide. This systematic review seeks to examine the prevalence of diabetes and its associated comorbid conditions, aiming to provide insights into the multifaceted impact of diabetes on a broader scale. DM exhibits a positive correlation with advancing age, and it's strongly influenced by genetic predisposition. In recent years, there has been a discernible global increase in the prevalence of type 1 diabetes (T1D), as evidenced by extensive epidemiological studies. Individuals with DM frequently have a positive familial history, and the presence of DM in both parents or solely the mother significantly amplifies genetic susceptibility. Moreover, non-genetic factors, such as acute psychological stressors, obesity, pregnancy, and smoking play a pivotal role in the development of DM. Notably, urinary tract infections (UTIs) are a common comorbidity in patients with type 2 diabetes (T2D) and all patients with T1D. T2D is prevalent, particularly among females, and its incidence rises with age. UTIs are prevalent among individuals with diabetes, particularly females, with Escherichia coli (E. coli) isolates being the primary etiological agents responsible for UTI inflammation. Insulin resistance is a common feature in both prediabetes and prehypertension, serving as a precursor to these conditions. The increasing incidence of T2D in regions with high tuberculosis (TB) prevalence emphasizes the significance of understanding DM as a substantial TB risk factor. DM is associated with a threefold elevation in TB risk and a twofold increase in unfavorable outcomes during TB treatment. Notably, the global prevalence of DM has led to a larger population of TB patients with comorbid DM than TB patients coinfected with HIV. Diabetes and sepsis contribute significantly to worldwide morbidity and mortality, with diabetic individuals experiencing more post-sepsis complications and increased mortality. The coexistence of hypertension and T2D is a common comorbidity, with hypertension incidence being twice as high among individuals with diabetes compared to those without, often linked to insulin resistance and a heightened risk of diabetes onset.

## Introduction and background

Diabetes, rather than being a singular ailment, encompasses a spectrum of disorders, primarily characterized by elevated blood glucose levels. A pivotal common pathway underlying numerous metabolic conditions is the occurrence of hyperglycemia. Among these, type 2 diabetes (T2D), accounting for 90-95% of diabetes cases, exhibits considerable heterogeneity in its etiological processes and associations with health outcomes. Recent data have unveiled the potential for classifying distinct subtypes of T2D using clustering techniques and clinical or genetic markers, each manifesting unique clinical features and distinct relationships with diabetic complications [1]. Diabetes mellitus (DM) has been linked to a myriad of comorbidities. Dysfunctional microbiota is known to exert far-reaching influences across various organ systems, heightening susceptibility to numerous diseases. Beyond endocrine system dysfunction, DM's ramifications extend to direct associations with cardiovascular and renal conditions. Moreover, DM exerts a profound impact on the immune system, rendering individuals more susceptible to infections and external aggressors, such as urinary tract infections (UTIs)[2].

The global management of tuberculosis (TB) is currently challenged by the escalating prevalence of T2D. An individual's risk of developing TB is tripled when afflicted with DM. Notably, the co-occurrence of TB and DM surpasses the incidence of TB-HIV coinfection [3,4]. The correlation between DM and TB was originally documented by the Persian philosopher Avicenna and has since garnered substantial attention in medical literature since the early twentieth century. Over the past three decades, the prevalence of DM in the global adult population has surged by 20%, and projections indicate that by 2040, the worldwide diabetic population will reach 642 million [5]. Significantly, the majority of these individuals (80%) reside in low- and middle-income countries, which coincidentally bear a heavy burden of endemic TB. Consequently, the World Health Organization has designated DM as a neglected yet substantial and emerging risk factor for TB. DM is responsible for approximately 28% of adult TB cases and a staggering 51% of TB cases in individuals aged 35 to 60, significantly eclipsing the contribution of HIV to TB incidence, which accounts for only 3-6% of TB cases among adults in the current era [6].

## Review

Methodology

**Figure 1:**
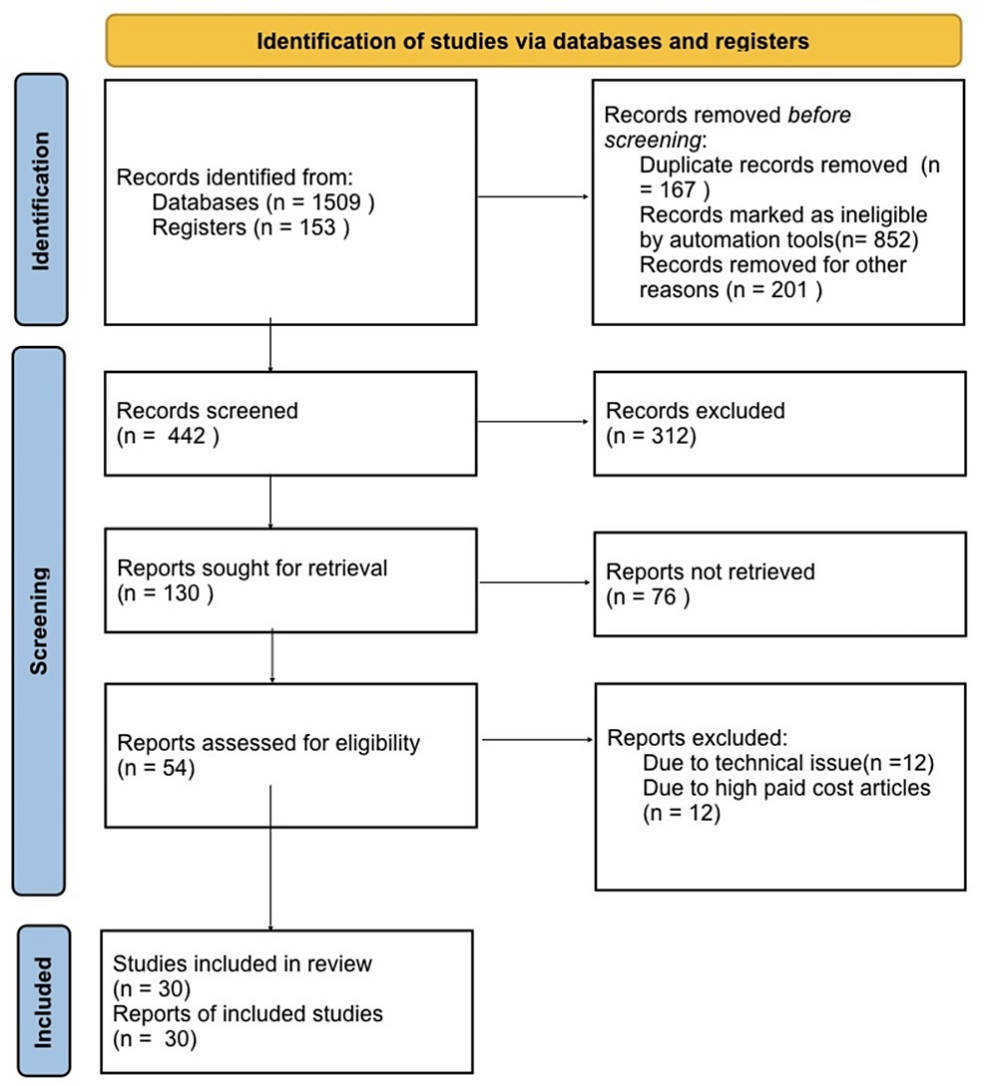
Search strategy utilised for the review.

In conducting this narrative review, we initiated our search process by accessing a database like 'PubMed.' Our search terms encompassed key terms ‘diabetes mellitus’ , ‘CKD’ , ‘HIV’, ‘DKD’, ‘TB’, ‘sepsis’ and ‘UTI’. We specifically focused on English-language publications. In cases where multiple reports stemming from a single study were identified in the literature, we prioritized the most recent one. Our inclusion criteria were designed to incorporate only review papers that introduced fresh insights and findings. Figure 1 shows the search strategy utilised.

DM as a risk factor for TB

The rising prevalence of diabetes globally can be attributed to several factors, including aging populations, urbanization, dietary shifts, and reduced physical activity [7]. This surge in diabetes incidence has also been closely linked to an increasing prevalence of obesity. Currently, an estimated 415 million cases of DM are distributed predominantly across 80% of the world's low- and middle-income countries [3].

However, the complex interplay between TB and DM, which has garnered substantial research attention, reveals substantial heterogeneity in study outcomes, with reported risk ratios spanning from 0.99 to 7.83 [3]. This heterogeneity underscores the inherent challenges in studying DM as a risk factor for TB, given the diverse characteristics of DM populations worldwide. These disparities encompass variations in age demographics, healthcare accessibility, glycemic control, and the presence of diverse types and severities of DM complications and medications. Moreover, the confluence of DM with other host characteristics can further elevate the risk of TB in affected individuals. This phenomenon is observed in cases such as type 1 diabetes (T1D) coupled with smoking, the presence of micro- and macrovascular complications, and the socioenvironmental conditions in which T1D patients reside [8,9]. The imperative to conduct comprehensive studies on DM and other host characteristics using multivariable analyses is evident, as this approach ensures the robustness and validity of research findings [9].

DM as a risk factor for kidney diseases

Individuals with diabetes may exhibit an increased susceptibility to non-specific renal diseases, which manifest as a gradual decline in renal function. Among these renal ailments, diabetic kidney disease (DKD), clinically known as diabetic nephropathy, stands out prominently. Diabetic nephropathy is characterized by the progressive deterioration of renal function, predominantly initiated by elevated blood glucose levels and frequently accompanied by the presence of albuminuria [10].

It is pertinent to note that several risk factors unrelated to diabetes, including hypertension, obesity, and dyslipidemia, can significantly contribute to the decline in renal function observed in diabetic individuals. The diagnostic criteria for DKD typically involve the assessment of elevated levels of albuminuria and a reduced estimated glomerular filtration rate (eGFR). These clinical indicators are instrumental in identifying the presence and severity of DKD. A critical aspect in understanding and categorizing DKD lies in considering the timing of diabetes diagnosis in relation to the onset of kidney disease. This temporal relationship plays a pivotal role in distinguishing between two distinct forms of DKD: diabetes-specific DKD and non-specific DKD. This differentiation allows for a more precise understanding of the underlying mechanisms and risk factors involved in renal complications associated with diabetes.

DM as a risk factor for cardiovascular disease

The term "cardiovascular disease" (CVD) encompasses a spectrum of disorders that affect both the heart and the blood vessels. While it is widely acknowledged that individuals with diabetes face an elevated susceptibility to CVD, our understanding of the underlying pathophysiological mechanisms linking these two conditions remains limited [11].

People with diabetes experience a significantly higher risk, ranging from two to tenfold, of developing various cardiovascular events or diseases when compared to those without diabetes [11]. These events include but are not limited to coronary heart disease (CHD), myocardial infarction, heart failure, and stroke [12]. It is essential to consider several additional risk factors that contribute to CVD among individuals with diabetes. These factors encompass additional microvascular complications like nephropathy or diabetic retinopathy, as well as variables such as gender, age, BMI, glycemic control, and HbA1c levels. Furthermore, assessing blood pressure levels and a history of smoking is of paramount importance, as they exert a substantial influence on the incidence of cardiovascular complications in individuals with diabetes [11].

DM as a risk factor for hypertension

The global increase in sedentary lifestyles and excessive calorie consumption has led to a rising prevalence of obesity and T2D, with a notable impact in developing and low-income nations. Projections indicate that by the year 2040, the number of T2D cases may surge to between 415 million and 642 million [13]. Concurrently, hypertension, characterized by elevated blood pressure, is also on the rise in several countries, currently affecting approximately 1.39 billion individuals worldwide [14]. It is essential to recognize that despite their potential ease of diagnosis at the bedside, T2D and hypertension are distinct conditions, each exhibiting its unique and intricate set of symptoms. Moreover, both conditions significantly heighten the risk of CVD, which carries a substantial mortality rate. The frequent co-occurrence of T2D and hypertension is not merely coincidental; rather, it stems from shared pathophysiological features, particularly those associated with obesity and insulin resistance. For instance, the San Antonio Heart Study revealed that a considerable percentage of T2D participants had hypertension by the age of 50, with hypertension detected in 85% of T2D individuals [15]. Conversely, approximately 50% of those with hypertension displayed impaired glucose tolerance or had received a T2D diagnosis [15].

Diabetes is intricately linked to both macrovascular and microvascular complications [16]. Microvascular complications involve small arteries and capillaries, while macrovascular complications affect major arteries, such as conduit vessels. The onset of diabetic vascular problems is significantly influenced by chronic hyperglycemia and insulin resistance. Multiple mechanisms, including increased formation of advanced glycation end-products (AGEs) and subsequent activation of the receptor for AGEs (RAGE), contribute to these complications, forming the AGE-RAGE axis. Emerging research also suggests that microRNAs (miRNAs) may play a role in diabetic vasculopathy development, with oxidative stress and inflammation serving as significant contributors to this process [17,18].

Hypertension, a major risk factor for diabetes-related vascular complications, is notable for its inherent vascular dysfunction and propensity to cause vascular injury. Table 1 lists the commonly used drugs for hypertension and diabetes.

**Table 1:** Commonly used medications for diabetes and for hypertension ACE: Angiotensin-converting enzyme ; DPP: Dipeptidyl peptidase

Medication for Diabetes	Medication for hypertension
Metformin	ACE inhibitor
Sulphonylureas	Calcium channel blockers
Alpha-glucosidase inhibitor	Thiazide type diuretics
DPP-4 inhibitors (gliptins)	Angiotensin receptor blockers
Thiazolidinediones (glitazones)	Beta blocker

DM as a risk factor for UTIs

The prevalence of DM, a metabolic and endocrine disorder, is on the rise. This condition, characterized by elevated blood sugar levels or hyperglycemia, results from abnormal insulin secretion and/or action. A significant proportion of patients seeking treatment for DM in primary care and family medicine clinics originate from Western and industrialized countries, where the disease is particularly widespread [19]. Recent data from the 2017 Global Burden of Diseases survey indicate that approximately 462 million individuals worldwide have been diagnosed with T2D, constituting about 6.2% of the global population [20]. The prevalence varies across age groups, affecting 4.4% of individuals aged 15 to 49, 15% of those aged 50 to 69, and 22% of those over 70 years old [20]. Notably, individuals with T2D are predisposed to various infections due to the urinary system's heightened susceptibility to infection [21-24].

Clinicians should possess a comprehensive understanding of the spectrum of UTI symptomatology in individuals with diabetes, as the predicted point prevalence rate of T2D is 6,059 per 10,000 people [25]. This profile encompasses a range of symptoms, including asymptomatic bacteriuria, cystitis, pyelitis, pyelonephritis, and urosepsis, with rarer and more severe conditions such as emphysematous pyelonephritis, cystitis, renal abscess, and renal papillary necrosis [26,27]. Screening for UTIs in diabetic patients should be a top priority, given the association of immune system dysfunctions, including humoral, cellular, and innate immunity impairments, with UTI development in diabetics. This proactive approach helps reduce the risk of diabetic renal complications by promptly identifying and treating bacteriuria. As hyperglycemia progresses, it leads to significant nerve and blood vessel damage, potentially resulting in CVD, cerebrovascular accidents, renal disorders, visual impairment, diabetic neuropathy, periodontal infections, diabetic foot problems, and, in severe cases, limb amputation [28,29]. This preventive strategy is pivotal for preserving renal health in individuals with diabetes, as it delays the onset of severe renal damage and mitigates the risk of renal failure. It is crucial to acknowledge that diabetes is associated with reduced immune function, glycosuria (excretion of glucose in urine), and bladder dysfunction, all of which serve as potential risk factors for UTIs [30,31]. T2D, a multifactorial condition characterized by reduced insulin production and low insulin sensitivity, is renowned for chronic hyperglycemia. Metabolic abnormalities, such as obesity and dyslipidemia associated with T2D, as well as inflammation and insulin resistance resulting from chronic and stress-induced hyperglycemia, collectively exacerbate the host's immune response to infections.

DM as a risk factor for sepsis

The major causes of illness and mortality in the world are diabetes and sepsis. Notably, diabetes raises the risk of post-sepsis sequelae, which helps explain why mortality rates are rising. In sepsis patients with diabetes, immune mechanisms that are dysregulated greatly enhance the host response. It is yet unclear how diabetes affects sepsis-related mortality, despite the widely held view that inadequate glycemic management contributes to a significant number of severe infections, insulin, metformin, and thiazolidinediones may lower the incidence and fatality rate of sepsis. According to some data, long-term exposure to high glucose levels may encourage immunological adaption, which would lower the death rate in diabetics with sepsis [32]. Sepsis also harms the immune system by shortening its lifespan and decreasing its ability to produce and operate. Over time, this disruption disturbs the immune system's delicate balance. The molecular network that leads to worse clinical outcomes in patients with sepsis and T2D remains unknown. Patients have undesirable effects as a result of these drugs' immune system-damaging effects [33,34].

Findings from multiple studies are listed in Table 2.

**Table 2:** Findings of the review articles studied TB: Tuberculosis; DM: Diabetes mellitus; T2D: Type 2 diabetes; UTI: Urinary tract infection; CVD: Cardiovascular disease

Study Title and Authors	Year	Key Findings/Recommendations
Ottmani SE, et al. [1]	2010	Meeting summary and recommendations on TB and DM.
Ronacher K, et al. [2]	2015	Focus on acquired immunodeficiencies related to TB, including HIV/AIDS and DM.
Jeon CY, Murray MB [3]	2008	Diabetes increases the risk of active TB based on a systematic review of observational studies.
Magliano DJ, Boyko EJ [4]	2021	Provides data on global diabetes prevalence and trends from the IDF Diabetes Atlas.
Hu F [7]	2011	Discusses the role of diet, lifestyle, and genetic factors in the globalization of diabetes.
Reed GW, et al. [8]	2013	Investigates the impact of diabetes and smoking on mortality in TB patients.
Kuo MC, Lin SH, et al. [9]	2013	Reveals that T2D is an independent risk factor for TB, based on a nationwide study.
Martínez-Castelao A, et al. [10]	2015	Discusses changes in the concept and epidemiology of diabetic nephropathy.
Earle K, et al. [11]	1992	Explores familial clustering of CVD in insulin-dependent diabetes patients with nephropathy.
Tuomilehto J [12]	1998	Examines the incidence of CVD in type 1 diabetic subjects with and without diabetic nephropathy.
Ogurtsova K, da Rocha Fernandes JD [13]	2017	Provides global estimates for diabetes prevalence in 2015 and projections for 2040 from the IDF Diabetes Atlas.
Mills KT, Bundy JD, Kelly TN [14]	2016	Analyzes global disparities in hypertension prevalence and control based on population-based studies from 90 countries.
Mitchell BD, Stern MP, et al. [16]	1990	Identifies risk factors for cardiovascular mortality in Mexican Americans and non-Hispanic whites.
Brownlee M [17]	2005	Discusses the pathobiology of diabetic complications and a unifying mechanism.
Zhang Y, Sun X, Icli B, et al. [18]	2017	Highlights the emerging roles of microRNAs in diabetic microvascular disease and their potential as therapeutic targets.
American Diabetes Association [19]	2010	Provides criteria for the diagnosis and classification of DM.
Khan MAB, Hashim MJ, et al. [20]	2020	Describes the epidemiology of T2D and global burden based on the Global Burden of Disease data.
Alemu M, Belete MA, et al. [21]	2020	Investigates bacterial profiles and associated factors of UTIs in diabetes patients.
Shah BR, Hux JE [23]	2003	Quantifies the risk of infectious diseases for people with diabetes.
Nath T, Das SK, Hazra S [24]	2021	Studies the pattern of uropathogens and antibiotic sensitivity in diabetes patients with UTIs.
Lim JH, Cho JH, et al. [26]	2013	Examines risk factors for recurrent UTIs in kidney transplant recipients.
Hooton TM [27]	1990	Discusses the epidemiology of UTI and the concept of significant bacteriuria.
Wukich DK, Raspovic KM, et al. [28]	2018	Indicates that patients with diabetic foot disease fear major lower-extremity amputation more than death.
Eren MA, Güneş AE, et al. [29]	2020	Examines the role of platelet-to-lymphocyte ratio and neutrophil-to-lymphocyte ratio in predicting the length and cost of hospital stay in patients with infected diabetic foot ulcers.
Lature LH, Lature ML, et al. [30]	2020	Assesses UTIs among type 2 diabetic patients in a rural teaching hospital.
Kumar R, Kumar R, et al. [31]	2019	Provides the clinical and microbiological profile of UTIs in diabetic versus non-diabetic individuals.
Tiwari S, Pratyush DD, et al. [33]	2011	Discusses the risk of sepsis in diabetes and its implications.
Frydrych LM, Fattahi F, et al. [34]	2017	Explores the relationship between diabetes and sepsis, including risk, recurrence, and outcomes.

## Conclusions

DM is a prevalent metabolic and endocrine disorder that continues to exhibit a rising prevalence globally. It is characterized by the elevation of blood sugar levels, termed hyperglycemia, resulting from dysregulations in insulin secretion and/or function. This condition is particularly prominent in Western and industrialized nations, where a substantial number of individuals seek medical care for DM. The prevalence of DM is on an upward trajectory, with nearly 500 million cases worldwide, making it one of the most widespread health issues in the developing world. DM not only impacts blood glucose levels but also exposes the body to a myriad of associated health issues, many of which are on the rise globally. Individuals with DM face an increased susceptibility to various illnesses and infections, notably UTIs. Additionally, they are more prone to comorbid conditions, primarily hypertension. The global incidence of obesity and T2D are also on the rise, driven by the widespread adoption of sedentary lifestyles and excessive caloric consumption. The frequent coexistence of these two conditions within the same individual is not coincidental but rather attributed to shared elements in their pathophysiology, particularly those associated with obesity and insulin resistance. Furthermore, the presence of DM and other host characteristics can amplify the risk of TB among individuals with diabetes. This heightened risk is evident in cases involving DM coupled with smoking, as well as the presence of micro- and macrovascular complications associated with diabetes, and even the social environment in which DM patients reside. Dysregulated immune pathways, a common observation in both sepsis and diabetes, play a substantial role in exacerbating the host response in diabetic patients afflicted with sepsis.
